# Ninth Annual Meeting of the Mississippi Valley Association—Held Feb., 17 and 18, 1853

**Published:** 1853-04

**Authors:** 


*Ninth Annual Meeting of the Mississippi Valley Association*—Held 17th and 18th Feb., 1853.

Persuant to adjournment, the society met in the lecture-room of the Ohio Dental College, at 10 A. M., the President, Dr. Taft in the chair.

Present, Drs. Bonsall, Jas. Taylor, Ulrey, McCullom, Watt, Hamill, Allen, Leslie, Goddard, and Griffith.

Executive Committee being called upon, presented through their chairman, Dr. Ulrey, a report, embracing the order of business, viz:

*Report of Executive Committee.*—The Executive Committee respectfully offer the following suggestions, as the order of business:

1. Report of Examining Committee.
2. Report of Officers.
3. Reports of Standing Committees.
4. Addresses and Essays.
5. Miscellaneous Business.—1st. Best Method of cleaning teeth and removing the "green stain," deep seated, from the teeth; also, the best article to be used for that purpose.

2d. Safest manner of extracting teeth.

3d. When the nerves of teeth are destroyed, is it proper to fill the fang and crown, or the crown only?

4th. Best method of destroying nerves in roots, in which pivots are to be inserted.

5th. Best method of inserting pivot teeth.

6th. Are compound fillings admissible in any condition of the teeth?

7th. The best metals for casts.

8th. The best method for making atmospheric pressure plates.

9th. The experience of gentlemen present, in the use of asbestos, for filling the bottoms of cavities in tender teeth,

J. P. ULREY,
DR. WATT.

On motion of Dr. Taylor, this report was accepted and adopted.

On Motion of Dr. Taylor, the president was requested to fill the vacancy in the examining committee, made by the abscence of Dr. Berry.

The chair appointed Dr. Bonsall on said committee.

The Treasurer presented reports, showing a balance of $114.26, in his hands.

On motion of Dr. Taylor, the chair appointed Drs. Ulrey and Hamill, an auditing committee.

On motion of Dr. Watt, the president was requested to deliver the introductory address, after the reading of which, it was on motion of Dr. Ulrey, requested for publication in the Dental Register.

In pursuance of the order of business, Dr. Watt read an essay.

On motion of Dr. Leslie, a copy was requested, for publication in the Dental Register of the West.

On motion of Dr. Taylor, it was resolved that Drs. Smith, H. E. Peebles, J. C. Ross, and Willard, be invited to take seats as corresponding members; also, that the dental class be invited to be present at the meeting of the society.

Dr. Allen arose, and after alluding to the fact of action having been had during the last meeting of the society, which he believed was aimed at him, stated, briefly, his position on the subject of patents—contended that this society was mistaken, and that the sentiments of nineteen-twentieths of the profession were with him.

Dr. Leslie, in consideration of the absence of Dr. Allen during the action of patents, and to afford others a chance to reply to him, and give Dr. Allen a liberal opportunity to state his views, moved the adoption of the following:

*Resolved,* That a committee of three be appointed, to take into consideration the course of Dr. Allen, on the subject of patents, and report to-morrow.

The question being taken on the adoption of the above, it was lost, "because of the time it would take, and which could be more profitable employed, and as Dr. Allen had been already heard, in vindication of himself."

On motion, adjournment to meet at 2, P. M.

---

AFTERNOON SESSION.

Meeting was called to order by the president. Present, Drs. Griffith, Goddard, Hamill, Watt, McCullom, Ulrey, Allen, Bonsall, Taylor, Leslie, Peebles, W. B. Ross, J. C. Ross, Smith, and Willard.

Minutes of morning session read and adopted.

On motion of Dr. Goddard, Dr. Ayres, of Danville Ky., was invited to take a seat as a corresponding member.

Under the order of business, Dr. Hamill proceeded to read an essay on irregularities of teeth. Dr. Taylor moved the acceptance of the essay, and opened discussion on the same. Nearly every member present, took part in the endeavor to impart or elicit facts and principles bearing on the subject.

On motion of Dr. Goddard, its further consideration was laid over until to-morrow morning, and the members invited to bring in specimens of cases treated by them.

Adjourned to meet at 7, P. M.

---

EVENING SESSION.

Minutes of afternoon session read and approved.

Present, Drs. Taft, Ulrey, Watt, Bonsall, Griffith, McCullom, Hamill, Ayres, Peebles, Goddard, Willard, J. C. Ross, Smith, and Leslie.

The subject of cleaning teeth and removing "green stain,"

was considered, during the main part of the evening session. It was discussed by Drs. Griffith, Allen, Bonsall, Ayres, Smith, Watt, Leslie, Ulrey, Peebles, and Taft.

The following query was presented by Dr. Goddard:

What is the application which can be made, to remove the sensibility occasioned by the use of the file in removing decay, or separating teeth.

This elicited the views of Drs. Goddard, Griffith, Allen, Peebles, Ulrey, and a recipe from Dr. Smith.

On motion adjourned to meet at 8½ A. M. to-morrow.

---

SECOND DAY—MORNING SESSION.

Present Drs. Taft, Bonsall, Griffith, M'Cullom, Hamill, Goddard, Peebles, Smith, Taylor, Allen, Watt, J. C. Ross, Ulrey, Ayres, and Leslie.

Minutes of previous evening's session read and adopted.

Dr. M'Cullom read a paper on the use of the file, which on motion was accepted.

A very interesting discussion, partaken in by the members generally, was had on the use of the file, which showed a general agreement as to its use and importance; speaking on the form of files, Dr. Leslie took occasion to present to the consideration of the association a separating file having a bevelled edge, a form which was first shown him by Professor Arthur, of Washington, by whom it was introduced to the profession.

Dr. Goddard moved that the order of business for 4 o'clock this afternoon, be the election of officers. Adopted.

On motion of Dr. Leslie it was

*Resolved*, That a standing committee, consisting of three, be appointed annually, styled The Committee on Dental Progress, whose duty it shall be to note all the improvements which may be made during the year, so far as it can ascertain them, with the names of the inventors and discoverers, and present the same in their report, which is also expected to present a general view of the *progress of dental science* for the current year.

The subject laid over from yesterday (inequalities of the teeth,) was now taken up, when the question on acceptance of Hamill's essay was renewed and adopted.

The following report was read, and on motion accepted:

The Examining Committee have had the names of Dr. Samuel Ayres of Danville Ky., Dr. James C. Ross of Nashville, Tennessee, and Dr. Samuel Ross, of Shelbyville Ky., presented to them for membership in this association, and take great pleasure, after due examination, in recommending them to your favorable consideration.

W. H. GODDARD.<br>CHAS. BONSALL, } *Examining Committee.*<br>H. M'CULLOM.

On motion of Dr. Goddard, a ballot was had on admitting the gentlemen presented by the Committee, to active membership in this society, after which the President announced them unanimously elected.

The second item under head of Miscellaneous business was now taken up and discussed, by Drs. Peebles, Goddard, Griffith, Taylor, and Allen.

On motion adjourned, to meet at 3½ P. M.

---

AFTERNOON SESSION.

Minutes of morning session read and approved.

Present, Drs. Taft, Bonsall, Watt, Ulrey, Goddard, Allen, Griffith, Ayres, Peebles J. C. Ross, M'Cullom, Hamill, W. B. Ross, Taylor, and Leslie.

The third item under head of Miscellaneous business was taken up and discussed by Drs. Griffith, Allen, Ayres, Peebles, Goddard, and Leslie, showing a greater variety of opinion on this than on any other subject discussed.

The society next went into an election of officers. After balloting the following officers were declared elected.

*President*—CHAS. BONSALL,<br>*Vice President*—SAM'L. AYRES,<br>*Recording Secretary*—A. M. LESLIE,

*Corresponding Sec'y.*—W. H. GODDARD.

*Treasurer*—C. BONSALL.

*Examining Committee*—Drs. Taft, Ulrey, and Jas. Taylor.

*Executive Committee*—Drs. J. C. Ross, Griffith, and Ayres.

On motion adjourned to meet at the office of Dr. Bonsall, at 7½, P. M.

---

EVENING SESSION.

Present Drs. Goddard, Taft, Peebles M'Cullom, Smith Hamill, Allen, Ulrey, Ayres, Watt, Bonsall, Taylor, and Leslie.

Minutes of afternoon session approved.

The President announced as the committee on Dental Progress; Drs. Allen, Ayres, and J. C. Ross.

Dr. Watt presented the following resolutions, which on motion, were adopted.

*Resolved,* That the association will award fifty dollars to the author of the best essay (not to be published,) on Dental Surgery, *adapted to general circulation.* Said essay, to be approved of by a committee, and to contain not less than fifty nor more than seventy-five pages duodecimo.

*Resolved,* That a committee of three be appointed to examine the essays presented, and report at next meeting. Essays competing for said award to be forwarded to the chairman of the committee as early as January 1, 1854.

On motion Drs. James Taylor, Cincinnati, O., W. H. Goddard Louisville, Ky., and J. Taft, Xenia, O., were appointed on essays under the foregoing resolutions.

On motion the Corresponding Secretary was requested to procure the publication of the resolutions on essays, in all the dental periodicals.

Dr. Taylor moved the appointment of a committee to examine the forceps presented under the resolution of the September meeting.

The chair appointed Drs. Goddard, Ayres and Bonsall said committee.

On motion the remaining items of miscellaneous business were laid on the table.

Dr. Griffith presented an under impression frame sent on by Dr. W. C. Ross of Shelbyville, Ky.

Dr. Taft read a paper describing an "improved mode of forming sockets, for gum teeth on plates."

On motion of Dr. Allen, the thanks of the society were voted to Dr. Taft for presenting the improvement, and a copy of the paper requested for publication in the Register.

Drs. Ayres, J. C. Ross and Ulrey were appointed to read essays at the next annual meeting of the society.

The committee on extracting instruments presented the following report.

*To The Mississippi Valley Association of Dental Surgeons:*

The committee appointed to examine a set of extracting instruments presented for the award offered at your last meeting, would respectfully report:

That we feel bound in justice to ourselves, and the association we represent, to say, as but one set was presented (and that not perfected,) although there are many good instruments among them, they consider some very defective, in form and mode of application, and there being no competition we are not willing to decide upon their merits.

W. H. GODDARD.
SAM'L. AYRES.
CHAS. BONSALL.

On motion report was accepted, and committee discharged.

On motion of Dr. Watt the report was adopted.

On motion of Dr. Goddard it was

*Resolved,* That this association will award twenty dollars, or a medal of equal value, on the most perfect set of extracting instruments, the same to be first approved of by the society.

On motion the Treasurer was authorised to pay the Janitor of the Dental College $3 for services rendered.

On motion *Resolved*, That the minutes of this meeting be published in the Dental Register.

Dr. Ulrey now moved, that the minutes as now read be adopted. Carried.

The society, together with several members of the medical profession proceeded to President Bonsall's dining-room, and spent some time in the discussion of subjects, in which great harmony of action was exhibited, and diverse tastes gratified.

On motion of Dr. M'Cullom,

*Resolved*, That the thanks of the association be tendered Dr. Bonsall for his hospitalities to the society.

On motion adjourned to meet at the Ohio College of Dental Surgery at 10 A. M. Wednesday February 24, 1854.

